# Soft Hydrogel Inspired by Elastomeric Proteins

**DOI:** 10.1021/acsbiomaterials.1c00817

**Published:** 2021-10-22

**Authors:** Antonietta Pepe, Lucia Maio, Angelo Bracalello, Luis Quintanilla-Sierra, Francisco Javier Arias, Alessandra Girotti, Brigida Bochicchio

**Affiliations:** † Laboratory of Bio-inspired Materials, Department of Science, University of Basilicata, Via Ateneo Lucano 10, 85100 Potenza, Italy; ‡ BIOFORGE CIBER-BBN, LUCIA Building, University of Valladolid, Paseo de Belen 19, 47011 Valladolid, Spain; § Smart Devices for NanoMedicine Group, LUCIA Building, University of Valladolid, Paseo de Belen 19, 47011 Valladolid, Spain

**Keywords:** elastin, hydrogel, circular dichroism, cytocompatibility, antiadhesive materials

## Abstract

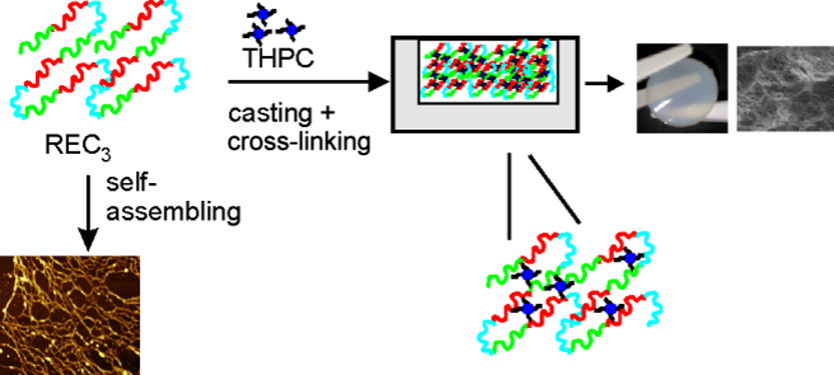

Elastin polypeptides based on -VPGVG- repeated motifs are widely used in the production of biomaterials because they are stimuli-responsive systems. On the other hand, glycine-rich sequences, mainly present in tropoelastin terminal domains, are responsible for the elastin self-assembly. In a previous study, we have recombinantly expressed a chimeric polypeptide, named resilin, elastin, and collagen (REC), inspired by glycine-rich motifs of elastin and containing resilin and collagen sequences as well. Herein, a three-block polypeptide, named (REC)_3_, was expressed starting from the previous monomer gene by introducing key modifications in the sequence. The choice was mandatory because the uneven distribution of the cross-linking sites in the monomer precluded the hydrogel production. In this work, the cross-linked polypeptide appeared as a soft hydrogel, as assessed by rheology, and the linear un-cross-linked trimer self-aggregated more rapidly than the REC monomer. The absence of cell-adhesive sequences did not affect cell viability, while it was functional to the production of a material presenting antiadhesive properties useful in the integration of synthetic devices in the body and preventing the invasion of cells.

## Introduction

Polypeptides inspired by proteins generally constitute the matrices, usually known as scaffolds, used in tissue engineering and regenerative medicine. The ongoing strategy is to implant *in situ* the scaffold seeded with cells able to trigger new tissue formation. In that case, the presence of adhesion sites for promoting cell adhesion and spreading is very common.^1^ Recombinant protein-inspired polypeptides offer the advantage of being highly biocompatible, with controlled molecular weight and low immunogenicity; therefore, they are generally preferred to natural polymers (alginate, chitin, and chitosan), synthetic polymers such as poly(ε-caprolactone) and polylactide, and copolymers such as poly(lactide-*co*-glycolide) and poly(lactide-*co*-ε-caprolactone).^2^ Elastomeric proteins are widespread in living organisms from invertebrates (resilin in insects, byssus in mussels, and abductin of bivalve mollusks) to mammalians (elastin and titin present in connective tissues and vertebrate muscles).^3^ Recently, resilin, present in arthropods, has captured attention for its outstanding mechanical properties and rubber-like characteristics.^4,5^ Beyond resilin, elastin is well-known in the field because the conventional design of elastin polypeptides (ELPs) is usually based on [Val-Pro-Gly-Xaa-Gly] pentapeptide motif *n*-fold repeated, where Xaa could be any amino acid except proline.^6^ This repeating motif, abundant in the central region of the tropoelastin molecule, exhibits intrinsic elasticity at the molecular level and is responsible for the inverse phase transition behavior acting as stimuli-responsive systems.^7−10^ However, human tropoelastin is rich in glycine-rich sequences with the Xaa-Gly-Gly-Zaa-Gly motif containing Val and/or Leu as guest residues. These regions are mainly present in the tropoelastin terminal domains and are responsible for elastin self-assembly.^11−13^ Previous results on (XGGZG)_3_ model peptides have shown that the residue type (X, Z = Val, Leu) and the exact position occupied by the residue in the motif are crucial in driving the self-aggregation and in defining the morphology of the formed nanostructures as well.^14^ Accordingly, glycine-rich polypeptides are emerging as an alternative class of elastin-inspired polypeptides in the design of recombinantly expressed polypeptides for biomedical applications.^15−18^ Recombinant elastomeric polypeptides were commonly enriched with adhesion sequences in order to improve cell invasion and spreading; however, adhesion to tissues could be detrimental in clinical practice, causing severe pain and inflammation as postsurgery complications.^19^ Several biomaterials, such as polyethylene glycol, were developed as active methods to prevent postsurgery adhesion, working mainly as a physical barrier.^20^ Very few examples were described where elastomeric proteins or derivatives were used as antiadhesive biomaterials.^21^ The aim of the present work is the design and production of an elastomeric protein-inspired hydrogel characterized by softness and elasticity and devoid of cell-adhesive sequences to be proposed as an antiadhesive material.

**Figure 1:**
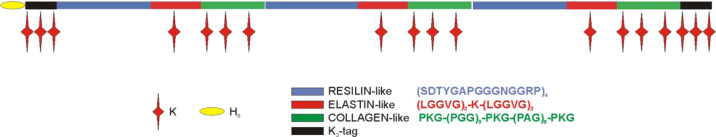
Schematic representation of the primary structure of the (REC)_3_ polypeptide.

Hydrogels in nature are three-dimensional structures naturally capturing water, while synthetic hydrogels are instead the cross-linked product of a polymer. Therefore, one of the main tasks to address is the use of mild conditions during the cross-linking reaction that are able to mimic at the best of the possibilities the physiological environment in terms of temperature, pressure, and *milieu*. Herein, a high-molecular-weight (HMW) chimeric polypeptide inspired by resilin, elastin, and collagen (REC)-like repeated motifs and named (REC)_3_ was engineered and characterized. It can be considered a “trimer” because it contains the REC monomer three-fold repeated. It was engineered starting from the monomer sequence gene codifying the REC polypeptide.^17^ However, the low solubility, the low molecular weight, and an uneven distribution of the cross-linking sites precluded the hydrogel production, limiting the analysis of the REC to the linear un-cross-linked polypeptide. Nevertheless, the un-cross-linked REC monomer showed the ability to self-assemble into a network of fibrils mimicking the extracellular matrix. Furthermore, Young’s modulus calculated to have 0.1–3 MPa values was typical of elastin- and resilin-inspired proteins.^3,22^ On that basis, the REC chimeric polypeptide was considered a good candidate as a biomaterial. The cartoon reported in Figure 1 schematizes the primary structure of the (REC)_3_ polypeptide.

The (REC)_3_ trimer shows ameliorative elements in the primary structure; *in primis*, it shows two lysine tags bound to N- and C-terminal residues that on one side introduced a higher number of cross-linking sites for cross-linking reactions and on the other side enhanced the solubility in aqueous solutions.

Un-cross-linked and cross-linked (REC)_3_ polypeptides were studied. The un-cross-linked polypeptide was investigated at the molecular level by circular dichroism (CD) and Fourier-transform infrared spectroscopy (FTIR) techniques and at the supramolecular level by electron microscopy. The propensity of the trimer to self-aggregate into fibrils was assessed by transmission electron microscopy (TEM) and atomic force microscopy (AFM) time-course studies. The (REC)_3_ polypeptide formed a hydrogel after cross-linking. Rheology analysis characterized the polypeptide as a soft hydrogel. Adhesion and cell viability assays were carried out.

## Materials and Methods

### (REC)_3_ Gene Construction and Biosynthesis

Genetic engineering techniques were performed to construct the REC multimeric gene, as previously reported. Briefly, the restriction and modification enzymes, when not differently indicated, were purchased from Fisher Scientific (Thermo Fisher Scientific, Waltham, MA, USA); the DNA ladder was purchased from Invitrogen; and DNA purification and plasmid extraction were performed with a PureLink Extract (Invitrogen, ThermoFischer, Waltham, MA, USA) and a Quantum Prep plasmid miniprep kit (BIO-RAD, Hercules, CA, USA), respectively. The polymerase chain reaction (PCR) site-directed mutagenesis was carried out using the primer *Sap*IRECfor and *Ear*IRECrev amplification (Table 1).

**Table 1:** Melting Temperature, Sequence, and Number of Mismatched Nucleotides with Respect to the Original Sequence of Oligonucleotides Used for Mutagenesis

primer	*T*_m_ (°C)	sequence	mismatches
*Sap*IREC for	74	5′GTCGAGCTCTTCAGTATCGGATACCTATGGCGCTCCTGGC 3′	6
*Ear*IREC rev	76	5′-CAGGCCTCTTCGTACGCCTTTGGGCCCTGCTGGGCCG-3′	6

Due to the DNA template composition, the Pfu DNA polymerase was substituted by the DNA polymerase Herculase II Fusion (Agilent Technologies, Santa Clara, CA, USA) for its ability to amplify difficult/GC-rich DNA. The desalted PCR product was digested by *Ear*I endonuclease and cloned in a modified pDrive vector (Qiagen, Hilden, Germany), and the correctness of the sequence of the monomer gene was verified by automatic sequencing. After gene cloning, the monomer gene *Ear*I was digested, isolated, and subjected to concatenation (Figure S1A), and the resulting products were cloned in a pDrive vector. The gene size and sequence were confirmed in diagnostic digestions with the restriction enzyme *Eco*RI (Figure S1B) and DNA automatic sequencing. The genes selected were sequentially subcloned into two modified pET expression vectors (Novagen, Podenzano, Italy) pET 10 6K and pET 14 H8 (Figure S1A,B). The *Escherichia coli* BL21 (DE3) expression strain was used for the bioproduction of the (REC)_3_ polypeptide, which was carried out as previously described.^17^ (REC)_3_ purification was carried out by immobilized metal-ion affinity chromatography (IMAC) in a HisTrap HP (GE healthcare, Chicago, IL, USA) affinity purification system according to the manufacturer’s instructions. Then, the purified (REC)_3_ polypeptide was dialyzed in cold ultrapure water and then lyophilized. Finally, the (REC)_3_ polypeptide was further purified by semipreparative reverse-phase high-performance liquid chromatography (RP-HPLC) on a Shimadzu automated HPLC system supplied with a semipreparative (250 × 10 mm, 5 μm) Jupiter C5 column (Phenomenex, Torrance, CA, USA). Eluents were A (0.1% TFA in H_2_O) and B (0.1% TFA in CH_3_CN). A binary gradient from 5 to 60% B was used. The final yield of the purified (REC)_3_ polypeptide is 10 mg/L of the bacterial culture. The (REC)_3_ polypeptide was characterized by proton nuclear magnetic resonance spectroscopy (^1^H NMR) using a Varian AV-400 (Agilent Technologies, Santa Clara, CA, USA).

### CD Spectroscopy

CD spectra were recorded at 0, 25, 37, and 60 °C. The concentration of the protein solution was 0.1 mg/mL (2.6 microMolar, *M*_W_ 37 760 Da) in PBS (10 mM phosphate-buffered saline, pH 7.0) and in 2,2,2-trifluoroethanol (TFE) using a J-815 spectropolarimeter (Jasco, Tokyo, Japan), equipped with a Haake thermostat (Thermo Fisher Scientific, Waltham, MA, USA), averaging 16 scans. Baselines were corrected by subtracting the solvent contribution. Cylindrical, fused quartz cells of 0.1 cm path length (Hellma, Müllheim, Germany) were employed. The data are expressed in terms of [θ]_MRW_, the mean residue ellipticity (degree–square centimeter per decimole) value, in order to compare the data obtained for different peptide lengths. TFE was purchased from Romil (Waterbeach, Cambridge, UK). Deionized water was purified using a Milli-Q reagent-grade water system from Merck Millipore (Milano, Italy).

### Fourier-Transform Infrared Spectroscopy

The spectra were recorded on 460 PLUS FTIR spectrometer (Jasco, Tokyo, Japan) using a resolution of 2 cm^–1^ and 256 scans and then smoothed by using the Savitzky–Golay algorithm. The sample was analyzed in D_2_O (50 mg/mL) by using CaF_2_ cells of 50 μm path length. The decomposition of FTIR spectra was obtained using the peak fitting module implemented in the Origin Software (MicroCalc Inc., Northampton, MA, USA) using the second derivative method. In the curve fitting procedure, the Voigt peak shape has been used for all peaks. The Voigt shape is a combination of the Gaussian and Lorentzian peak shapes and accounts for the broadening present in the FTIR spectrum.

### TEM Time-Course Studies

The (REC)_3_ polypeptide (0.5 mg) was solubilized in ultrapure water and incubated at 37 °C for 0, 24, and 72 h. Twenty microliters of the solution were deposited onto carbon-coated copper grids. Negative staining was performed by applying few drops of 1% uranyl acetate in ultrapure water used to increase the contrast and the electron density of the samples. After air-drying, grids were observed by a G^2^ 20 Twin transmission electron microscope (Fei Tecnai, Hillsboro, OR, USA) operating at 100 kV.

### AFM Time-Course Studies

The samples were solubilized in ultrapure water (concentration of 0.5 mg/mL) and incubated at 37 °C for 7 days. Every 24 h, an aliquot (10 μL) of the incubated sample was withdrawn and deposited as drops on silicon (100) wafer substrates (Aldrich, Saint Louis, Mo, USA). After air-drying at room temperature, the AFM images were obtained by using the XE-120 microscope (Park Systems, Suwon, South Korea) in air and at room temperature (Figure S2a). Data acquisitions were carried out in the intermittent contact mode at scan rates between 0.4 and 0.7 Hz, using rectangular Si cantilevers (NCHR, Park Systems, Suwon, South Korea) having the radius of curvature of less than 10 nm and with the nominal resonance frequency and force constant of 330 kHz and 42 N/m, respectively. AFM images were elaborated by using Gwyddion 2.22 software (http://gwyddion.net). The resolution enhancement was obtained by applying the local contrast procedure. The method is useful for visualizing features in areas with low and high variations of the *z*-axis at the same time.

### Image Analysis

Processing of the AFM images was carried out using the free AFM Gwyddion software (https://www.gwyddion.net). One hundred measurements were carried out, and the results are plotted in Figure S2. Height measurements were obtained from scan sizes of 5 × 5 μm or less by measuring height profiles normal to the fibril axis (Figure S2b). Heights values were obtained by averaging the measurements from a large number of individual fibrils (>90), with errors being calculated as one standard deviation (1σ) from the mean.

### Hydrogel Formation

To a solution containing the (REC)_3_ polypeptide (0.75 μmol in 250 μL) was added 0.53 μL (3.7 μmol) of the tetra-armed cross-linker tetrakis(hydroxymethyl)phosphonium chloride solution (THPC) (Sigma-Aldrich, Saint Louis, Mo, USA) (80% in H_2_O, *d* = 1.341 g/L, *M*_w_ 190.56). Ultrapure water was added to a final volume of 1 mL. The polymer/cross-linker molar ratio was 1:4.5 (1:1 THPC to ELP reactive group molar ratio). The reaction was carried out at pH 10, 25 °C for 2.5 h and poured into customized Teflon molds (Ø: 13.5 mm; *h*: 2 mm). The hydrogel was stable at room temperature and used for rheological measurements and morphological analysis.

### Scanning Electron Microscopy

In order to reduce at a minimum the formation of large water crystals, the hydrogels were rapidly frozen by an ultra-low-temperature freezer operating at −80 °C before freeze-drying (lyophilization). Afterward, it was dropped into liquid nitrogen and physically fractured. Images of lyophilized hydrogels were obtained by a JSM-820 scanning electron microscope (JEOL, Tokyo, Japan) operating at 6.0 kV with prior gold sputtering coating (Balzers-SCD 004, Liechtenstein) procedures.

### Rheology

Rheological measurements were carried out on the hydrogels obtained by the cross-linking reaction. Rheological experiments were performed using a strain-controlled AR-2000ex rheometer (TA Instruments, New Castle, DE, USA) with the hydrogel submerged in water. Cylindrical swollen gel samples were placed between parallel plates of nonporous stainless steel (Ø: 12 mm). In order to prevent gel slippage, an adequate normal force was applied. A gap of higher than 1000 μm was always used. Measurements were carried out at 37 °C, with the sample temperature being controlled and maintained using a Peltier device. Two measurements were performed in the shear deformation mode. First, the range of strain amplitudes over which the gels exhibited a linear region of viscoelasticity was determined. Thus, a dynamic strain sweep (with amplitudes ranging between 0.01 and 20%) was carried out at a frequency of 1 Hz to measure the dynamic shear modulus, G*, as a function of strain. Second, dynamic frequency sweep tests were performed to determine the dependence of the storage modulus, *G*′, and loss modulus, *G*″, on frequency. Specifically, a frequency sweep between 0.01 and 10 Hz at 1% strain (corresponding to the hydrogel linear region) was chosen. The complex modulus magnitude, |G*| [|G*|^2^ = (*G*′)^2^ + (*G*″)^2^], and the loss factor (tan δ, where δ is the phase angle between the applied stimulus and the corresponding response) were also obtained. Each measurement was performed in duplicate, and the average is shown in the rheological results.

### Cell Culture

Human umbilical vein endothelial cells (HUVECs, ref cc-2517) and the endothelial growth medium (EGM) were purchased from Lonza (Lonza Walker, Walkersville, MD, USA). Normal human adipose-derived mesenchymal stem cells (hMSCs, ref R7788-115), basal medium Dulbecco’s modified Eagle’s medium (DMEM), fetal bovine serum (FBS), penicillin streptomycin solution, trypsin–ethylenediaminetriacetic acid (EDTA), fibronectin, bovine serum albumin (BSA), Dulbecco’s phosphate-buffered saline (DPBS), and an Alamar Blue viability kit were supplied by Invitrogen (ThermoFischer, Waltham, MA, USA). hMSCs were cultured in DMEM supplemented with 100 U/mL penicillin, 0.1 mg/mL streptomycin, and 10% FBS at 37 °C under 10% CO_2_, and the medium was replaced every 2 days. HUVECs were expanded in complete EGM and sub-cultured when they are 70–85% confluent. Cells were incubated at 37 °C in a humidified atmosphere of 5% CO_2_, and the medium was replaced every 48 h. When required, cell harvesting and subculturing were performed using a solution of 0.05% Trypsin–EDTA.

### Cell Viability Assay

In viability experiments, cells in the complete medium were seeded at 10^4^ cells/cm^2^ in a 96-well black clear-bottom plate. After 16 h of incubation, unattached cells were removed, and cells were incubated for 24 or 72 h with the complete medium for untreated cells or the complete medium containing 5 mg/mL (REC)_3_ polypeptides for treated cells. Then, the cells were washed with DPBS. The metabolic activity was evaluated by an Alamar Blue assay, in which the cells were incubated in 100 μL of a 10% solution of Alamar Blue in complete media. After 2 h of incubation, the fluorescence intensity (F.I.) of aliquots of the test samples and controls were measured at an emission wavelength of 590 nm after excitation at 560 nm using a SpectraMax M2e microplate reader (Molecular Devices, San Jose, CA, USA). Cell viability analysis by a LIVE/DEAD viability/cytotoxicity kit for mammalian cells was carried out according to the manufacturer’s guidelines. Untreated cells were used as the viability positive control. Briefly, a stock solution of the LIVE/DEAD reagents (1 μM calcein AM and 2 μM EthD-1 in 10 mL of DPBS) was prepared, and 100 μL was added in each well. After incubation for 20 min in the dark, the F.I. emission was measured at 530 and 645 nm after excitation at 485 and 525 nm (SpectraMax M5e microplate reader). The percentage of viable cells was calculated following the equation
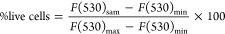
1where *F*(530)_sam_ is the fluorescence at 530 nm of the treated cells labeled with calcein AM and EthD-1; *F*(530)_max_ is the fluorescence at 530 nm of the untreated cells labeled with calcein AM only; and *F*(530)_min_is the background fluorescence corresponding to the fluorescence at 530 nm of the untreated cells labeled with EthD-1 only. Additionally, photographic images of cultures were taken using an Eclipse Ti-SR fluorescence microscope (Nikon, Tokyo, Japan). Three independent experiments, each condition at least in triplicate, were performed.

### Cell Adhesion Assay

The cell culture 24-well plates were coated with a solution of 1 mg/mL (REC)_3_, 1 mg/mL BSA, or 10 μg/mL human fibronectin for 2 h at 37 °C. The surfaces were washed three times with DPBS and blocked for 1 h in 1% BSA at 37 °C to prevent nonspecific adhesion of cells. Finally, the blocking solution was removed and the wells rinsed ready to be used in the adhesion assay. Near-confluence cells (passages 2––4) were enzymatically harvested in a short trypsin–EDTA treatment. They were then washed, resuspended in a serum-free culture medium, and seeded on the 24-well plate previously treated as described above at 10,000 cells/cm^2^ density. Before seeding, the viable cell counts were evaluated using a standard Trypan Blue exclusion assay (Thermo Fisher Scientific, Waltham, MA, USA). After 1 h of incubation, the minimal medium was replaced with the complete version. Each coated-type surface was tested in three independent experiments in triplicate, and images from nine randomly selected fields were taken 1 h after seeding.

### Statistical Analysis

Data are reported as mean ± SD (*n* = 3). Statistical analysis involved a variance analysis in combination with a subsequent analysis using the Bonferroni method. A *p*-value of less than 0.05 was considered to be statistically significant. Error bars represent the standard deviation of the mean. Data were handled using the GraphPad Statistics software version 6 (San Diego, CA, USA).

## Results and Discussion

### (REC)_3_ Biosynthesis

With the principal aim of improving the chimeric REC biopolymer performances in terms of bioproduction, cross-linking, chemical modification capability, and mechanical properties, a modified version was designed. The designed polypeptide, consisting of a three-fold repeated REC sequence, has a higher molecular weight and was enriched in lysine residues in order to increase its production yield, its solubility in an aqueous environment, and the number of reactive groups suitable for cross-linking. Therefore, in the first step, multimerization of the gene coding for the REC biopolymer using the seamless cloning method that involves the use of type IIS restriction endonucleases was performed.^7,23^ This type of endonucleases, as *Ear*I, recognizes asymmetric base sequences and cleaves DNA outside their recognition site, thereby cleaving any DNA sequence that is at a defined distance from their recognition sequence. The restriction sites of *Ear*I was introduced flanking the DNA codifying for the peptide monomer REC of 411 bp through PCR site-direct mutagenesis (Figure S3A). The amplification product *Ear*I was digested and cloned in a vector, and the positive clone containing the correct sequence was selected to be utilized for the seamless multimerization by the concatamerization method.^23,24^ As shown in Figure S3C, concatamerization was successful in obtaining dimers and trimers of REC, proving to be a useful tool for the one-step polymerization of monomer genes in a protein polymer biosynthesis approach. With the purpose of simultaneously improving the biopolymer solubility and reactivity, the content of lysine residues in the polypeptide sequence was also increased. However, the addition of lysine residues was not performed at the monomeric gene level but after multimerization as the insertion of polar amino acids inside the polypeptide sequence could perturb biopolymer mechanical and self-assembling features. Accordingly, the previously obtained monomer, dimer, and trimer genes have been subcloned into a modified plasmid whose codifying sequence includes three additional lysines at both N- and C-termini (Figures S1A and S4). The three (REC)*n* (*n* = 1–3) polypeptides were successfully expressed in *E. coli,* as shown in Figure S5, where the three polypeptides were expressed as major bands with respect to the endogenous bacterial proteins.

**Figure 2:**
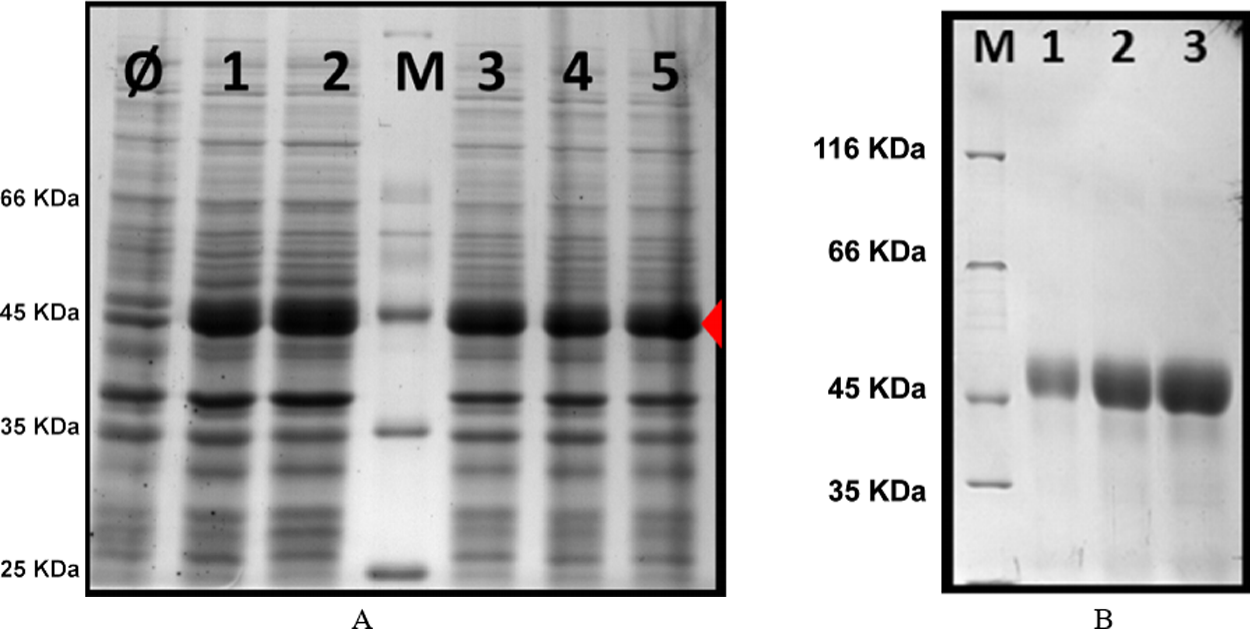
12% SDS-PAGE analysis of (REC)_3_ production screening (A) and purification process (B). (A) Total protein fraction analysis of *E. coli* BLR(DE3) after overnight induction in a modified TB medium. Lane M: protein marker; lane Ø—negative control, untransformed BLR(DE3); and Lanes 1–5, five transformed colonies were randomly selected and analyzed. The red marker highlights the (REC)_3_ polypeptide production by different transformants. (B) Increasing amounts of the purified (REC)_3_ polypeptide were evaluated: 5 μg (lane 1), 10 μg (lane 2), and 15 μg (lane 3); M: protein marker, the molecular weight of bands is indicated.

We can also observe that the (REC)_1–3_ polypeptides showed a higher molecular weight than theoretically expected. An electrophoretic mobility delay that determines an apparently higher molecular weight has been previously described in recombinant protein polymers whose composition in hydrophobic amino acids was around 80%, the same percentage of REC biopolymers.^15^ Finally, due to the good expression yield of (REC)_3_, its gene was subcloned in another modified pET expression vector (pET 14 H8) (Figures S1B and S4) to insert an octahistidine tag that allows its purification by IMAC. The obtained (REC)_3_ polypeptide is a 442-amino-acid polymer whose complete sequence is shown in Figure S6. Each monomer, three times repeated in the molecule, contains three different building blocks derived from resilin-, elastin-, and collagen-like motifs.^10^ The addition of lysine flanking the (REC)_3_ sequence increased the polypeptide solubility from 1 mg/mL for the REC monomer up to 100 mg/mL for the (REC)_3_ polypeptide. The octahistidine tag at the N-terminus facilitated polypeptide purification and thereafter could allow the detection in tissues of the (REC)_3_-containing devices through immunological techniques. The identity and purity of the (REC)_3_ polypeptide was confirmed by sodium dodecyl phosphate–polyacrylamide gel electrophoresis (SDS-PAGE) gels (Figure 2), amino acid analysis (Figure S7), ^1^H NMR (Figure S8), matrix-assisted laser desorption/ionization time of flight mass spectrometry (MALDI-TOF/MS, Table S1, Figure S9), and RP-HPLC analysis (Figure S10). The results of the characterization showed that the (REC)_3_ polypeptide was pure and monodisperse and that its composition and molecular mass matched the expected results.

### CD Spectroscopy Studies

**Figure 3:**
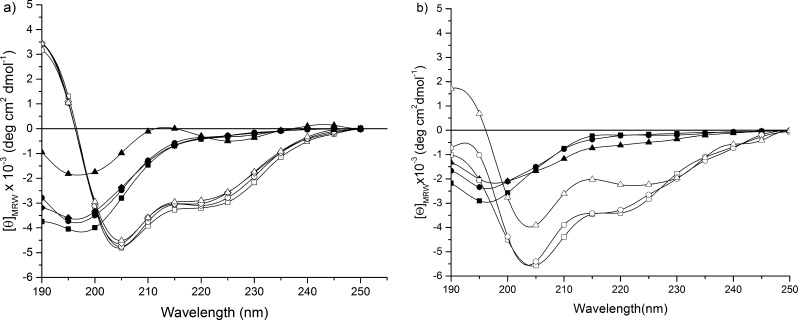
(a) CD spectra of the (REC)_3_ polypeptide in PBS (filled symbols) and TFE (open symbols) at the indicated temperatures: 0 °C (squares); 25 °C (circles); 37 °C (diamonds); and 60 °C (triangles); (b) CD spectra of the REC polypeptide in an aqueous solution (filled symbols) and TFE (open symbols) at the indicated temperatures: 0 °C (squares); 25 °C (circles); and 60 °C (triangles).

CD investigation was carried out on the linear (REC)_3_ polypeptide in different solvents and temperatures in order to gain insights into the secondary structure. In Figure 3a, the CD spectra of the (REC)_3_ polypeptide recorded at the indicated temperatures are shown.

At 0 °C, the CD spectrum recorded in phosphate-buffered aqueous solution (10 mM PBS, pH 7.0) shows a negative band at 198 nm. On increasing the temperature to 25, 37, and 60 °C, the band is gradually blue-shifted and reduced in intensity. Additionally, at 60 °C, a weak positive band appears at 213 nm together with a negative one at 226 nm. The strongly negative π–π* band at ∼198 nm is usually attributed to either unordered or PPII (poly-l-proline II conformation) conformations. However, the CD spectrum at 0 °C shows a negative band at [λ] = 200 nm having [θ] = 4000. The intensity is too weak to be exclusively assigned either to PPII or unordered conformations.^25,26^ This finding together with the reduction in intensity of the band with the increasing temperature could be indicative of the growing contribution from conformations with a positive ellipticity at 200 nm such as the β-turn, β-strand, and (less likely) α-helix.^27^

The hypothesis of the copresence of multiple conformations is strengthened by the lack in the CD spectrum at 0 °C of the positive, essentially n−π*, band at ∼216 nm expected for peptides with high amounts of the PPII structure very stable at low temperatures^11,28,29^ and by the absence of an isodichroic point. Summarizing, the polypeptide in phosphate-buffered aqueous solution is able to adopt flexible and folded structures in conformational equilibrium among them. In the CD spectra recorded in TFE, a less polar solvent, we observe a negative band at 204 nm, a shoulder at 222 nm, and a strong positive band at 190 nm. On increasing the temperature to 25, 37, and 60 °C, we observe the slight reduction of the negative band, while the positive one remains unchanged. These spectral findings could be assigned to the presence of a type I (III) of β-turns, stable at high temperatures, and a random coil. The conformational space is then populated by conformers expected for elastin glycine-rich regions as well as resilin sequences.^30^ For comparison, in Figure 3b, the CD spectra of a REC monomer polypeptide, containing an H_6_-histidine tag at the N-terminus, are shown. In a phosphate-buffered aqueous solution, the temperature does not substantially affect the curves. The curves exhibit negative peaks less intense than the corresponding observed for the trimer in the same conditions. These spectral findings suggest that the conformation of the REC monomer contains more random coils and less poly-l-proline II than the trimer. The CD spectra in TFE show only one curve, the one at 60 °C, with a slight positive band at about 200 nm, whereas, at the remaining temperatures, only negative bands are visible. This is indicative of the dominance of unordered conformations, especially at low temperatures, in comparison to the (REC)_3_ trimer.

### FTIR Studies

The decomposed spectrum of the amide I region of FTIR spectroscopy carried out in a D_2_O solution is shown in Figure S11. The main components appear at 1624, 1638, 1654, and 1673 cm^–1^ together with minor contributions at 1611, 1688, and 1699 cm^–1^. Generally, bands arising from aggregated strands are observed in the range 1608–1618 cm^–1^; therefore, the component at 1611 cm^–1^ is assigned to strands.^31^ The remaining two components could be assigned to vibrational modes of antiparallel β-sheet conformations.^32,33^ The components at 1624 and 1638 cm^–1^ correspond to the vibrational modes of hydrogen-bonded β-turn and β-pleated sheets, respectively.^34^ On the other hand, the O–H stretching mode from water is usually found in the range 1640–1650 cm^–1^. Herein, the spectrum was carried out in D_2_O solution, and the component at 1638 cm^–1^ is attributed to water.^35^ The component at 1673 cm^–1^ is assigned to the PPII conformation which is an extended structure lacking intramolecular hydrogen bonds.^36^ In fact, in the range 1660–1670 cm^–1^, the bands are usually assigned to non-hydrogen-bonded C=O groups or those weakly bonded to the solvent. The component at 1654 cm^–1^ is generally assigned to the contribution of the random coil and/or to the α-helix. However, herein, the α-helix was not revealed by CD studies. On this basis, we assign the component at 1654 cm^–1^ to a random coil. On summarizing, FTIR analysis showed for the polypeptide the presence of multiple conformations comprising an ensemble of β-turns, PPII, and random coils, as also suggested by CD studies.

### TEM Time-Course Studies

**Figure 4:**
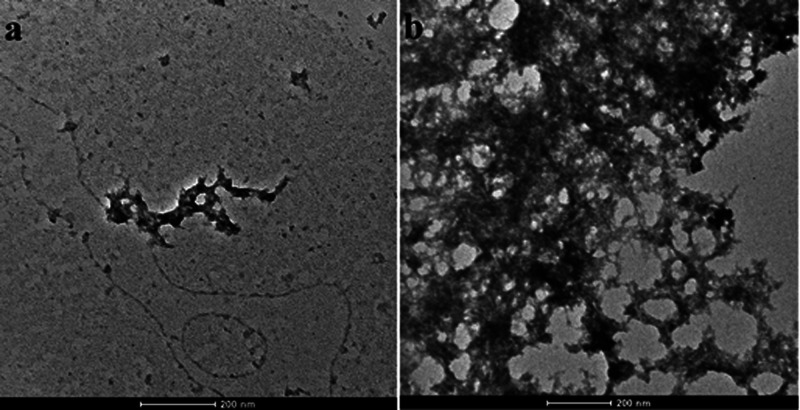
TEM micrographs of (REC)_3_ incubated at 37 °C after drop deposition (a) and after 24 h (b).

The propensity of the polypeptide to self-assemble in nanostructured aggregates was assessed by a TEM time course-study. The TEM images of the (REC)_3_ polypeptide are shown in Figure 4 at the indicated incubation times.

Immediately after the deposition of the drop on a metallic surface (*t*_0_), the early formation of a network is observed. After 24 h, an extensive mesh was detected. The morphology is similar to that found for the aggregate of α-elastin, as shown by TEM.^37,38^ This feature is important because it corroborates the hypothesis that protein-inspired polypeptides mimic efficiently the parent protein. Nevertheless, it is worthy of note that the monomeric REC incubated at the same temperature was able to self-aggregate only after 72 h^17^ The rationale for that could be found in the higher molecular weight of 37 kDa of the polypeptide *versus* 12 kDa of the monomeric REC, that increases the number of interactions among the polypeptide chains and the resulting entanglements.

### AFM Time-Course Studies

**Figure 5:**
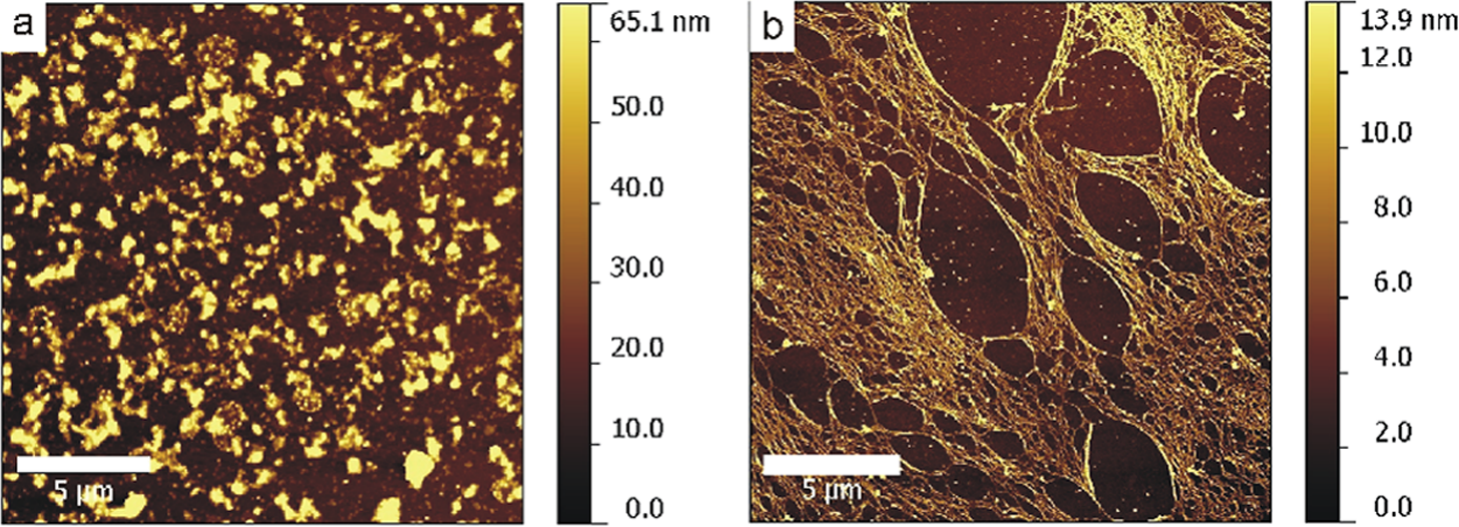
AFM images of (REC)_3_ incubated at 37 °C after 0 (a) and 24 (b) h.

The propensity of the (REC)_3_ polypeptide to adopt fibers having dimensions ranging from nanometers to micrometers was assessed by an AFM time-course study. In Figure 5, the AFM images of the (REC)_3_ polypeptide are shown at the indicated incubation times. Immediately after the deposition of the drop (*t*_0_), only small globules are present, whereas after incubation at 37 °C for 24 h (*t*_24_), a highly extended fibrillar mesh is visible. The intricate network is constituted by interlocked fibrils that form an expanded netting structure. The diameters of the nanofibrils were analyzed by measuring the height of the fibers by AFM (Figure S2a). The height distribution of 100 manually measured fibers from AFM images is shown in Figure S2b. The average height of the nanofibers is 6.8 ± 2.6 nm. We measured the height of the fibers instead of the diameter as the exact height of the nanostructures at the Z-range can be accurately measured by AFM, while the measurements of the lateral dimensions (*xy* plane) are affected by the error due to the tip convolution. In the same conditions (incubation at 37 °C for 24 h), the monomeric REC polypeptide did not form any organized nanostructure; only after 3 days of incubation were isolated nanofibrils discerned, whereas 7 days of incubation at 50 °C were necessary to observe a reticulated mesh structure.

### Hydrogel Studies

Glutaraldehyde is a well-known cross-linking reagent of elastin-like polypeptides containing lysine. The main disadvantage in the use of glutaraldehyde is the cytotoxicity toward cells.^39^ Hexamethylene diisocyanate is considered an alternative lysine-targeted cross-linker because its cytotoxicity is considered to be lower than that of glutaraldehyde.^40^ Lim *et al.* introduced β-[tris(hydroxylmethyl) phosphino] propionic acid (THPP) as a trifunctional cross-linker among lysine working at physiological pH and in aqueous solution.^41^ Generally, THPP cross-linked hydrogels are considered highly cytocompatible because fibroblasts and chondrocytes were successfully incorporated. In this study, we choose to use THPC as a cheaper alternative to THPP. It serves as a tetrafunctional cross-linker of lysine residues acting through a Mannich-type reaction and able to assure cytocompatibility toward cells.^42,43^

**Figure 6:**
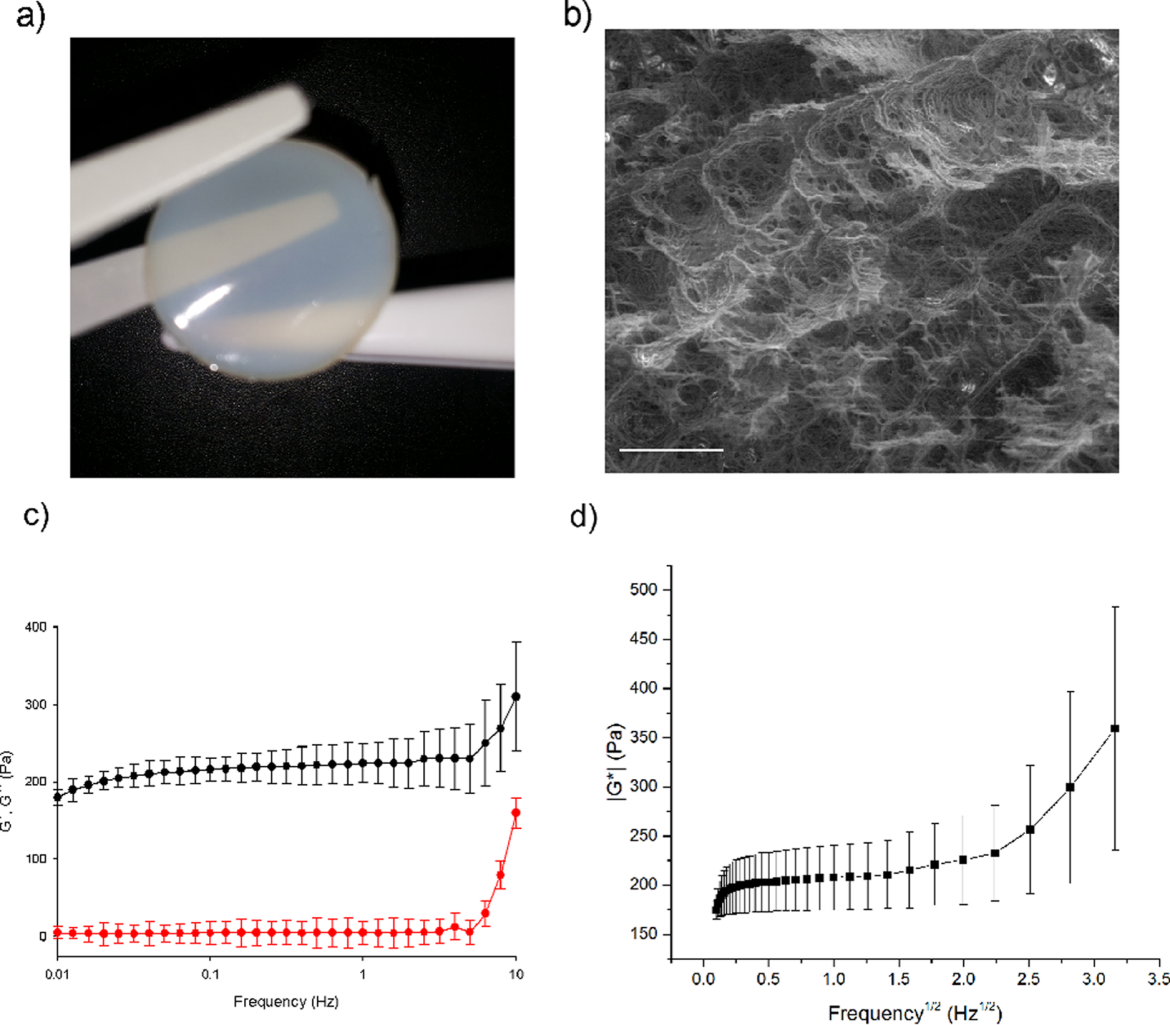
(a) Hydrogel; (b) SEM image of the hydrogel, where bar represents 30 μm; (c) evolution of *G*′ and *G*″ with frequency at 1% strain: average storage modulus (black) and loss modulus (red); and (d) dependence of the complex modulus magnitude with *f*^1/2^.

The (REC)_3_ polypeptide at a concentration of 50 mg/mL was cross-linked in a Teflon mold by THPC. The resulting hydrogel was shown in Figure 6a. At the microscopic level, the hydrogel appears as a sponge with interconnected pores (Figure 6b), even if the visualization of freeze-dried hydrogels by SEM could introduce an artificial porosity due to the formation of ice crystals during the freezing of the hydrogel.^44^ The hydrogel was further characterized by rheology. In primis, the linear viscoelastic region of the hydrogel was determined (Figure S12).^45,46^

A |G*| value of around 250 Pa was found for the 50 mg/mL hydrogel concentration, remaining independent of the strain amplitude (linear viscoelastic behavior) throughout the strain amplitude range considered (up to 20%) to ensure that rheological measurements were carried out within the linear viscoelastic region, all subsequent rheological tests were performed at 1% strain. In Figure 6c, the evolution of the storage, *G*′, and loss, *G*″, moduli with frequency have been represented. Only a slight increase in both *G*′ and *G*″ is observed for frequencies lower than 5 Hz. For higher frequencies, a significant increase is observed in both moduli. Focusing on 1 Hz, the values of the average storage and loss modulus are 226.2 ± 3.5 and 4.1 ± 1.9 Pa, respectively. The corresponding phase angle has an average value of 1.0 ± 0.5° at this frequency. The gel strength can be estimated from the magnitude of *G*′. According to the modulus value, the gel can be categorized as a soft hydrogel. The significant difference between *G*′ and *G*″ (*G*′ ≫ *G*″) indicates that the storage modulus is the major contributor to |G*|. Moreover, the quite low phase angles (even for 1.50°) correspond to highly elastic energy-storing hydrogels. With the aim to discuss the different physical mechanisms contributing to the viscoelastic behavior of the gel, the dependence of |G*| on *f*^1/2^ has been plotted in Figure 6d. Two different regions are observed: whereas a nonlinear relationship with *f*^1/2^ is found at low frequencies (around 0.1–0.14 Hz^1/2^), there is a frequency range in which a linear relationship is observed (roughly in the range 0.14–1.41 Hz^1/2^). This linear dependence is lost at frequencies higher than 1.41 Hz^1/2^. The first region corresponds to the intrinsic viscoelasticity (mainly dominated by the relaxation, reconfiguration, and conformational mobility of polymer chains), while the linear dependence on *f*^1/2^ indicates a poroviscoelasticity mechanism where the viscous drag of interstitial fluid through the porous (REC)_3_ polypeptide network and fluid–solid frictional interactions due to fluid pressurization dominate.^47−49^ By using a least-squares fitting of the experimental data in the linear region of Figure 6d, the slope can be calculated. This slope is related to the gel permeability that is a macroscopic measure of the ease with which fluid can flow through the matrix. Obviously, permeability decreases as the matrix becomes denser and more compact. In our chimera gel, a slope of around 20 Pa/Hz^1/2^ was estimated for the 50 mg/mL hydrogel concentration. In a previous work focused on click gels,^50^ a slope of about 200 Pa/Hz^1/2^ was obtained for a gel concentration of 50 mg/mL with an average pore size of units of microns. The low chimera gel slope indicates a quite high hydrogel permeability, in agreement with the significant average pore size (tens of microns) observed in the SEM micrographs (Figure 6b). On the basis of previous results, it has been shown that the (REC)_3_ polypeptide was designed to be more performant than the REC monomer and single blocks (elastin, resilin, and collagen). The results demonstrate that the properties of the trimer and its corresponding hydrogel in terms of physicochemical properties, mechanical characteristics, and self-assembly are significantly improved. The insertion of resilin sequences increased the hydrophilicity in comparison to that of the elastin or collagen counterparts, enhancing the solubility and triggering self-aggregation. As a matter of fact, the trimer was cross-linked in a hydrogel and aggregated irreversibly into a complex network of fibers in only 1 day at physiological temperature, while elastin peptides gave rise to reversible phase separation upon the coacervation process occurring at their transition temperatures depending on different variables such as the peptide molecular weight, molecular composition, and ionic force of the medium.

### Cell Viability and Adhesiveness of (REC)_3_

**Figure 7:**
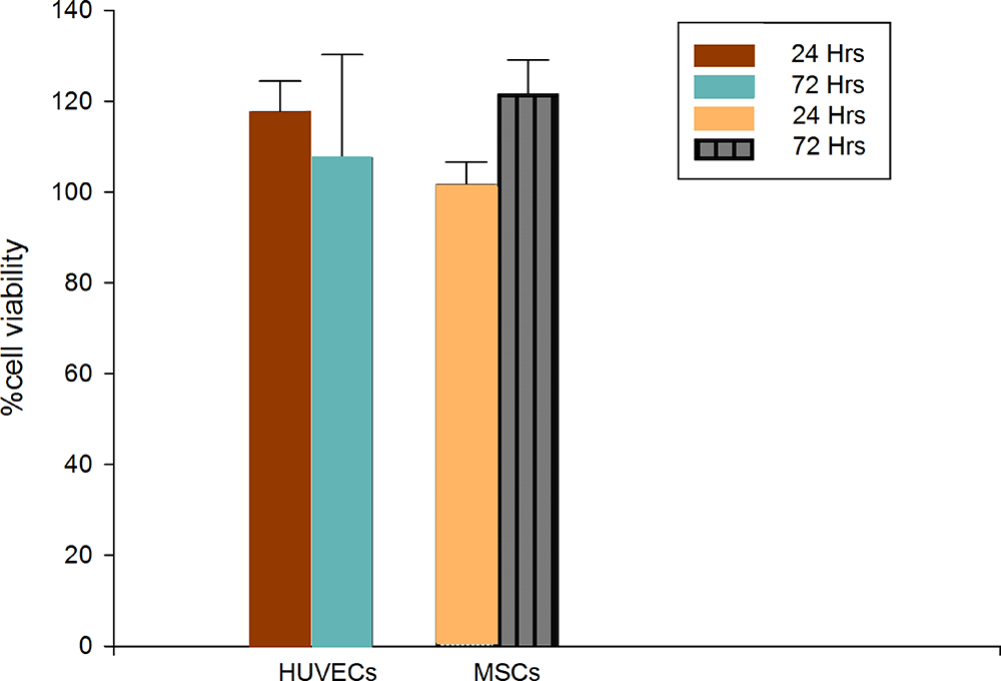
Viability percentage as the metabolic activity of HUVECs and MSCs with respect to untreated cells in the same conditions. Cells were incubated with 5 mg/mL (REC)_3_ for 24 or 72 h. Metabolic active cell numbers were measured, each sample four-fold, using the Alamar Blue assay kit in three independent experiments. No statistically significant differences are observed. The error bars represent the standard deviation.

In order to evaluate the ability to sustain the growth of different cell types by (REC)_3_-coated dishes, the development and phenotype of human primary cell cultures in the presence of (REC)_3_ was evaluated. The choice of primary cells instead of commonly used immortalized cell lines was dictated by the need to produce more physiologically relevant data. When dealing with materials whose purpose is to be in contact with any biological tissue, one of the first aspects to deal with is to confirm the absence of cytotoxicity in a suitable cell model that resembles the target tissue. First, we determined the cytotoxic effect of (REC)_3_ when endothelial and mesenchymal stem cultures were incubated in complete media with 5 mg/mL (REC)_3_ polypeptide for 1 or 3 days. In Figure 7, the results of the viability assays are reported as a measure of the metabolic activity of cell cultures.

After 24 h, the metabolic activity of HUVECs and MSCs incubated with 5 mg/mL (REC)_3_ polypeptide compared to that of untreated cells was 118 and 102%, respectively; while after 72 h of incubation time, the values of HUVECs and MSCs were 108 and 122%, respectively, suggesting that the metabolic activity of the cultures of both cell types is not affected by the presence of the (REC)_3_ polypeptide in the culture media.

Indeed, in all cases, we observe a greater viability than that of the untreated cells even if no significant differences between the analyzed time points were observed. Thus, we can conclude that under the experimental conditions used, the system does not cause a decrease in the viability of either mesenchymal stem or endothelial cells.

The sequence of (REC)_3_ was designed without cell-adhesion sequences to develop a biocompatible biomaterial presenting antiadhesive properties for the integration of synthetic devices in the body preventing the invasion of cells, for example, endothelial ones. To evaluate the cell adhesiveness, (REC)_3_–cell interactions with respect to cell cultures grown on negative (NC: the natural protein BSA) and positive controls (PC: an adhesive structural protein fibronectin) were assessed. *In vitro* studies of the adhesion and morphology were performed using both HUVECs and hMSCs. The early cell adhesion was analyzed after 60 min of seeding, the cells having been resuspended and incubated in a serum-free medium to avoid interference from serum compounds and complements in the specific adhesion mechanism. As shown in Figure S13, both cell types were grown on (REC)_3_-coated plates and on the adhesion negative control, and almost all cells presented a globular shape, being not anchored to the substrate; by contrast, cells grown on the positive control acquired a spread morphology, indicative of the high interaction of cells with the substrate. Differences in the morphology are clearer in the comparison of the early cell adhesion on positive (FN) and negative (BSA) controls. Analysis of the cell size, shape, and brightness showed that on the support that promotes cell attachment (fibronectin-coated positive control), the cells with the rounded morphology in suspension are flattening out on the substrate, thus increasing their cell areas and losing brightness. On the contrary, the cellular parameters observed for MSCs and HUVECs seeded on REC_3_ are very similar to those observed for the cells seeded on the negative control surface (BSA-coated), meanly brighter and rounder cells.

**Figure 8:**
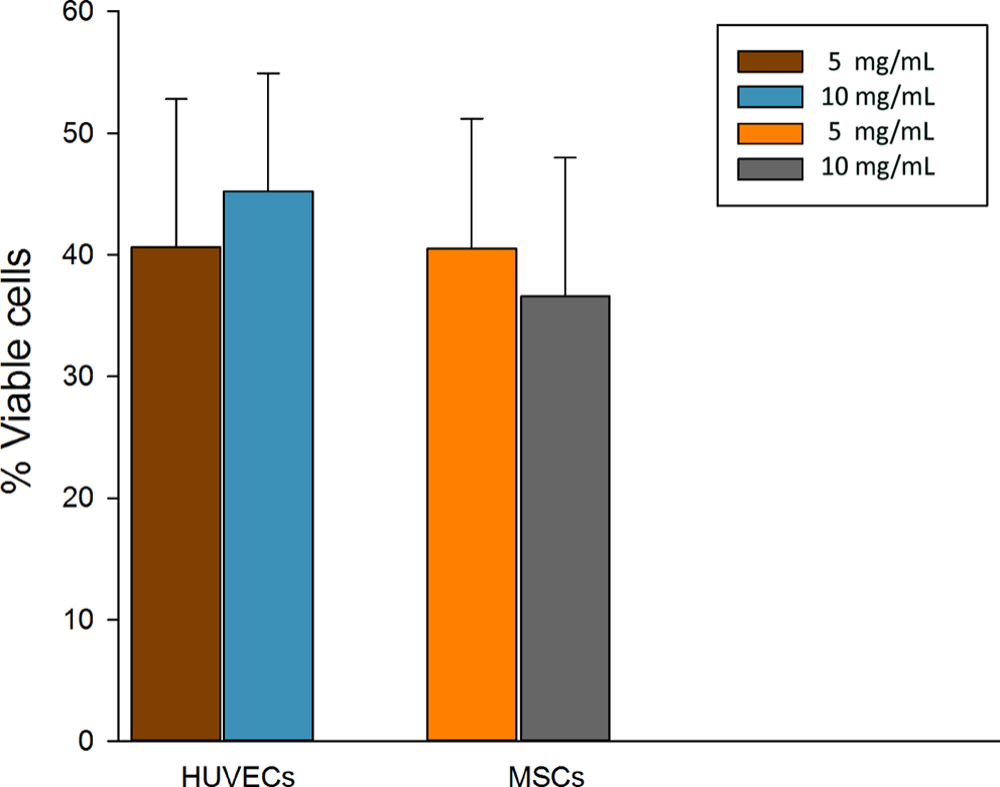
Viability of HUVECs and MSCs cultured on surfaces coated with (REC)_3_ solutions at 5 or 10 mg/mL concentrations. Histogram represents the percentages of live cells after 4 days of incubation with respect to live cells cultured on a standard tissue culture support. The error bars correspond to the standard deviation.

This result confirms the hypothesis that the (REC)_3_ sequence, being devoid of appropriate biochemical signals, is unable to promote early cell adhesion. Moreover, we have studied the viability and morphology of both cell cultures grown on surfaces coated with (REC)_3_ polypeptide solutions at 0, 5, or 10 mg/mL concentrations 4 days after seeding. In order to evaluate cell viability, the living cell number has been measured by a LIVE/DEAD assay (Figure 8).

In this case, we observed a reduction in the cell numbers with respect to the number of cells cultured on standard surfaces. The percentages of viable HUVECs were 45.2 and 40.6%, while the percentages of live MSCs were 40.5 and 36.6%, when cultured on (REC)_3_ surfaces coated at 5 or 10 mg/mL concentrations, respectively. Interestingly, no significant dose-dependent differences were observed when two different concentrations of (REC)_3_ were employed. These data suggest that the lower concentration (5 mg/mL) of the (REC)_3_ polypeptide is also sufficient to thoroughly cover the substrate. Further experiments on this aspect will be addressed by ongoing research.

**Figure 9:**
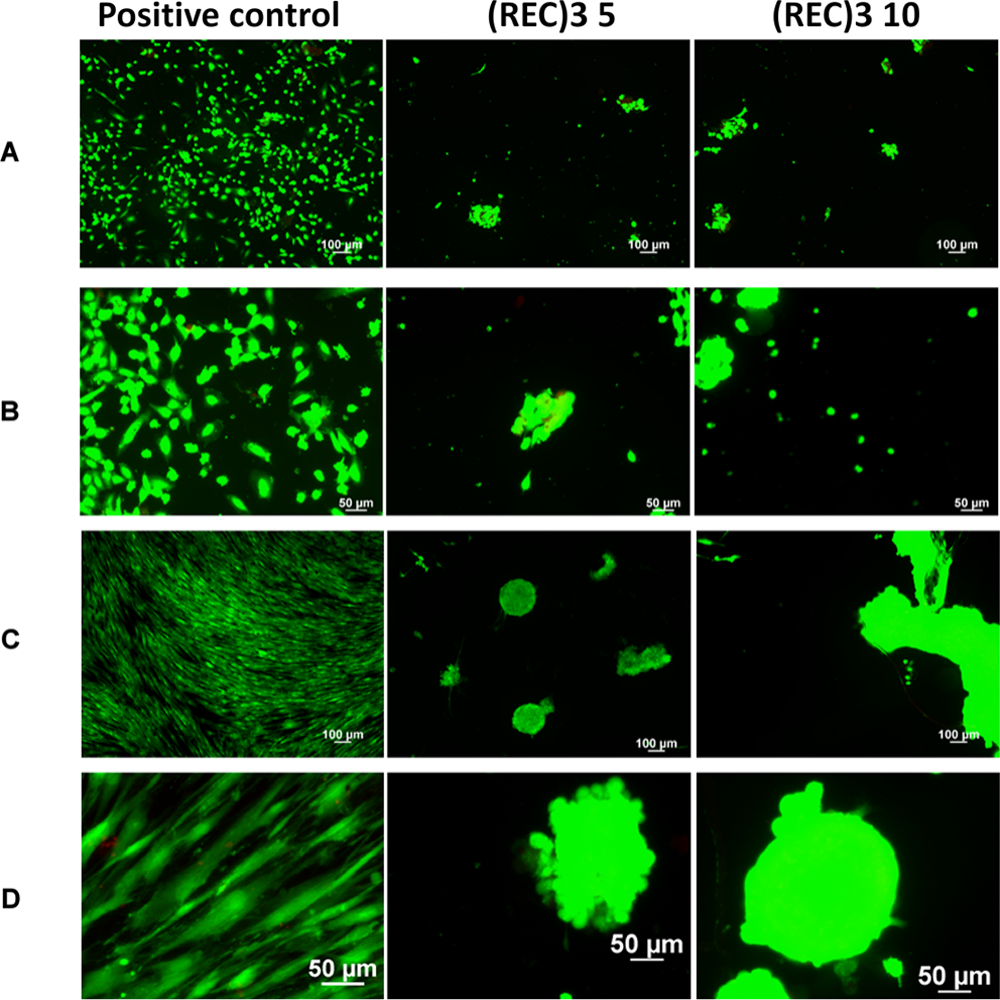
Panels A (10×) and B (20×): representative images of HUVECs cultured on fibronectin (positive control), 5 mg/mL or 10 mg/mL (REC)_3_-coated surfaces. Panels C (10×) and D (20×): representative images of hMSCs cultured on fibronectin (positive control), 5 mg/mL or 10 mg/mL (REC)_3_-coated surfaces. The living cells fluoresce green, whereas dead cells with compromised membranes fluoresce red.

With the aim of evaluating the cell morphology as a response of cells when deposited on (REC)_3_-coated plates, representative images of calcein/ethidium-stained cell cultures were taken and analyzed (Figure 9). The staining based on plasma membrane integrity and esterase activity is able to discriminate between live cells in green from dead ones in red. As shown in Figure 9, HUVEC and MSC cultures grown on the positive control have developed a well-spread morphology.

The cell number and morphology of HUVECs and MSCs seeded on (REC)_3_-coated surfaces were clearly different from those observed on the positive control. Only a few rounded cells and several aggregates composed mainly of viable cells were found, thereby suggesting that minor passive adhesion is the main cell–scaffold interaction. Even when cultured for 4 days, the cells were incapable of anchoring to the surface and grow in clusters, thus presenting a minimal contact area with (REC)_3_-coated surfaces. Although the presence of the components of the culture medium and the conditions of static culture have been prolonged, the (REC)_3_ polypeptide effectively inhibits cell adhesion. Commonly, cell antiadhesive properties are related to the hydrophilic/hydrophobic balance of the substrate. Both a highly hydrophobic substrate and a highly hydrophilic substrate are not suitable for cell adhesion. Interestingly, although the presence of the lysine residue increases the hydrophilicity of the biopolymer, it is still not sufficient to trigger cell adhesion. Probably, the conformational flexibility responsible for the cell antiadhesiveness predominates on specific electrostatic interactions. The cell antiadhesive properties of the (REC)_3_ polypeptide-coated surfaces could be rationalized taking into account thermodynamic aspects. As a matter of fact, we have shown by CD and FTIR spectroscopies that the (REC)_3_ polypeptide populates a high number of conformations, eliciting a high flexibility of the polypeptide chain. The high flexibility of the polypeptide chain ensures a high entropy state of the system that could prevent the attachment of cells. Probably, the adsorption of proteins, considered as the first step of cell adhesion, is also prevented for the same reasons.

Non-dhesive scaffold and nonfouling coatings may prevent some common complications during synthetic medical device integration, as well as scaffold-related infections and/or cell colonization when not required.

## Conclusions

In the present work, a HMW polypeptide containing three-fold repeated REC was produced and studied. At the molecular level, the trimer adopted flexible secondary structures composed of β-turns, unordered and PPII conformations quickly interconverting in a dynamic equilibrium extended-folded, the source of the high entropy of elastomeric protein in the relaxed state.^51,52^ At the supramolecular level, the linear (REC)_3_ polypeptide was able to rapidly self-aggregate in a mesh of nanofibrils. It took only 24 h in comparison to 72 h for the REC monomer. The increased solubility of the (REC)_3_ polypeptide (up to 100 mg/mL) due to the insertion of charged lysine prompted the production of a soft viscoelastic hydrogel by the cross-linking reaction. Furthermore, the (REC)_3_ trimer showed the absence of cytotoxicity together with noticeable cell antiadhesive properties. These results support the use of the (REC)_3_ polypeptide as antiadhesive materials, designed to prevent cell and tissue adhesion. On the other hand, the effectiveness of the (REC)_3_ polypeptide as a potential cell antiadhesive coating needs to be further evaluated *in vivo*. Studies are ongoing.

## Supporting Information Available

Gel electrophoresis; schematic representation of the subcloning strategy for the introduction of lysine residues in (REC)_*n*_ ends; sequence of the codifying region of pET10 6K and pET 14 H8 vectors; primary structure of the (REC)_3_ polypeptide; SDS-PAGE Coomassie Blue staining of total protein fractions of *E. coli* BL21(DE3) strain colonies after overnight induction in modified TB medium; amino acid analysis; 1H NMR spectrum; MALDI-TOF analysis; chromatogram of the (REC)_3_ polypeptide after HPLC purification; decomposed FTIR spectrum; AFM image of (REC)_3_ incubated at 37 °C after 24 h; height distribution of 100 fibers from AFM images of (REC)_3_; strain dependence of the complex modulus magnitude, |*G**|, at 37 °C for the (REC)_3_ hydrogel at a concentration of 50 mg/mL; phase-contrast images of mesenchymal and endothelial cells seeded on protein-coated surfaces; and images of HUVECs and MSCs cultured on fibronectin (positive control) or (REC)_3_-coated surfaces (PDF)

## Abbreviations

CD: circular dichroism
ECM: extracellular matrix
FTIR: Fourier-transform infrared spectroscopy
PPII: poly-l-proline II conformation
(REC): resilin, elastin, collagen monomer polypeptide
(REC)3: resilin, elastin, collagen trimer polypeptide
TEM: transmission electron microscopy
SEM: scanning electron microscopy
AFM: atomic force microscopy
HPLC: high-performance liquid chromatography
MALDI-TOF: matrix-assisted laser desorption/ionization time of flight
