# Supplementary material for: Soft Hydrogel Inspired by Elastomeric Proteins
Source: ACS Biomater Sci Eng. 2021 Oct 22;7(11):5028–38. doi: 10.1021/acsbiomaterials.1c00817 (PMC8579378; doi:10.1021/acsbiomaterials.1c00817)
Supplement: Supplementary file 1 — ab1c00817_si_001.pdf [file ab1c00817_si_001.pdf]

Supporting Information

Antonietta Pepe[a‡], *Lucia Maio*[a,b,], *Angelo Bracalello*[a‡], *Luis Quintanilla-Sierra*[b], *Francisco Javier Arias*[c], *Alessandra Girotti*[b]*, *Brigida Bochicchio*[a]*

[a] Laboratory of Bio-inspired Materials, Department of Science, University of Basilicata, Via Ateneo Lucano 10, 85100 Potenza (ITALY).

[b] BIOFORGE CIBER-BBN, LUCIA building, University of Valladolid, Paseo de Belen 19, 47011 Valladolid (SPAIN).

[c] Smart Devices for NanoMedicine Group, LUCIA building, University of Valladolid, Paseo de Belen 19, 47011 Valladolid (SPAIN).

‡ These authors contributed equally.

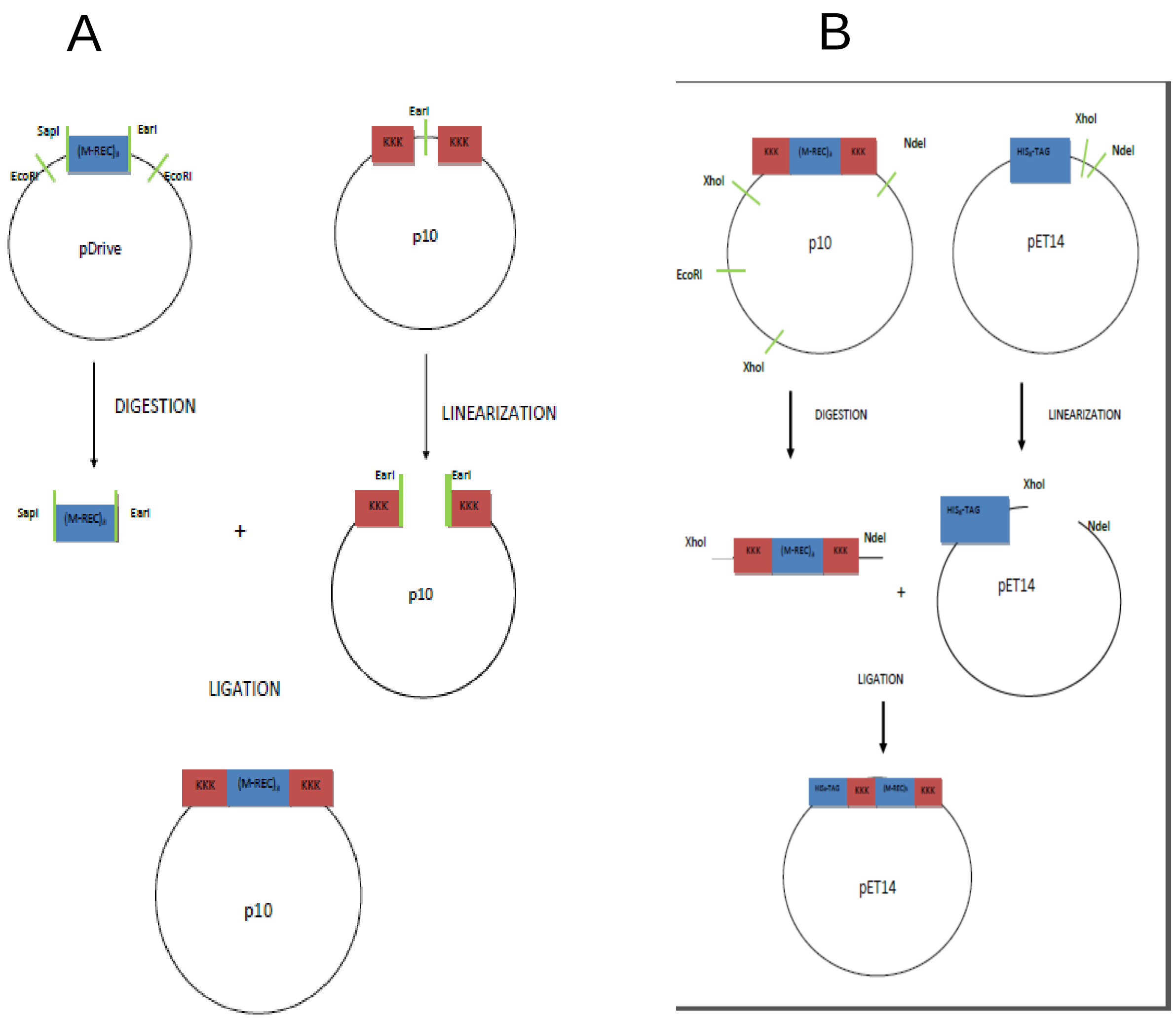

Figure S1. Schematic representation of sub-cloning strategy for introduction lysine residues in $(REC)_n$ ends (A) and the final $(REC)_3$ gene (B).

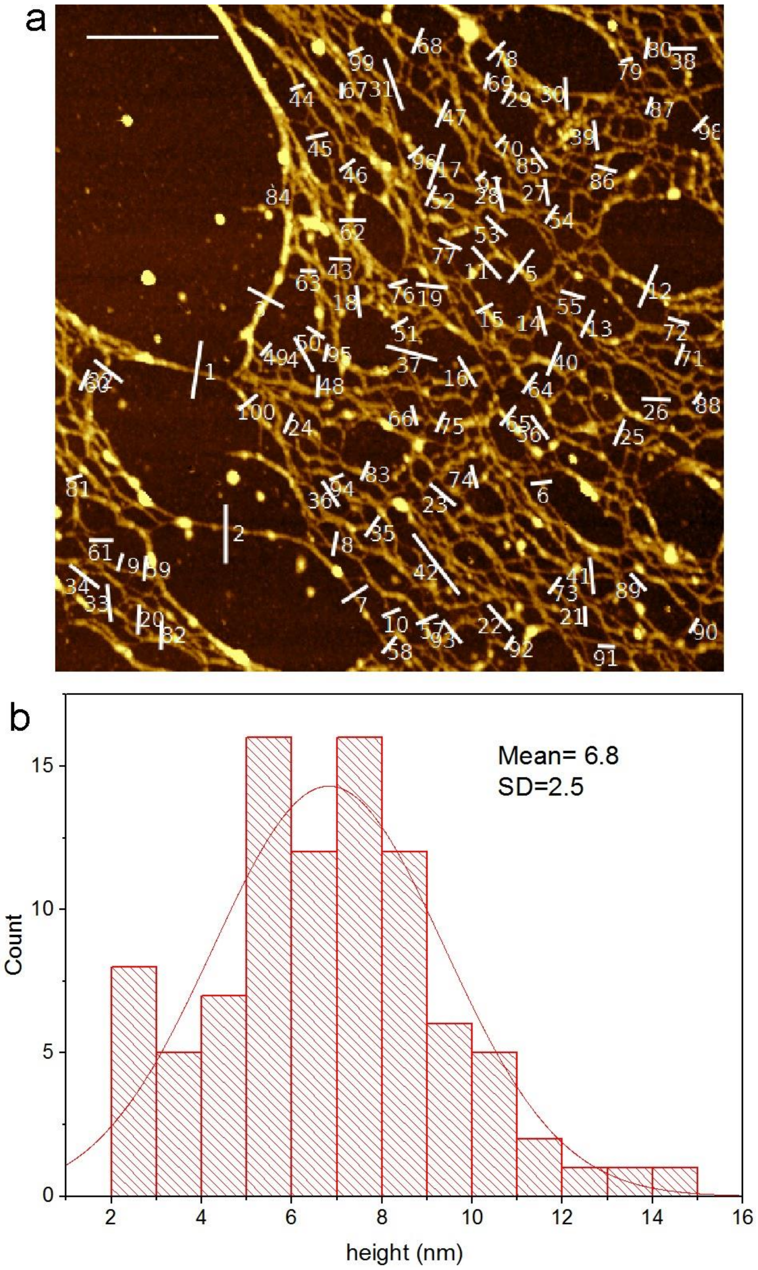

Figure S2. a) AFM image of $(REC)_3$ incubated at 37°C after 24 hours; b) Height distribution of 100 fibers from AFM images of $(REC)_3$ incubated at 37°C after 24 hours.

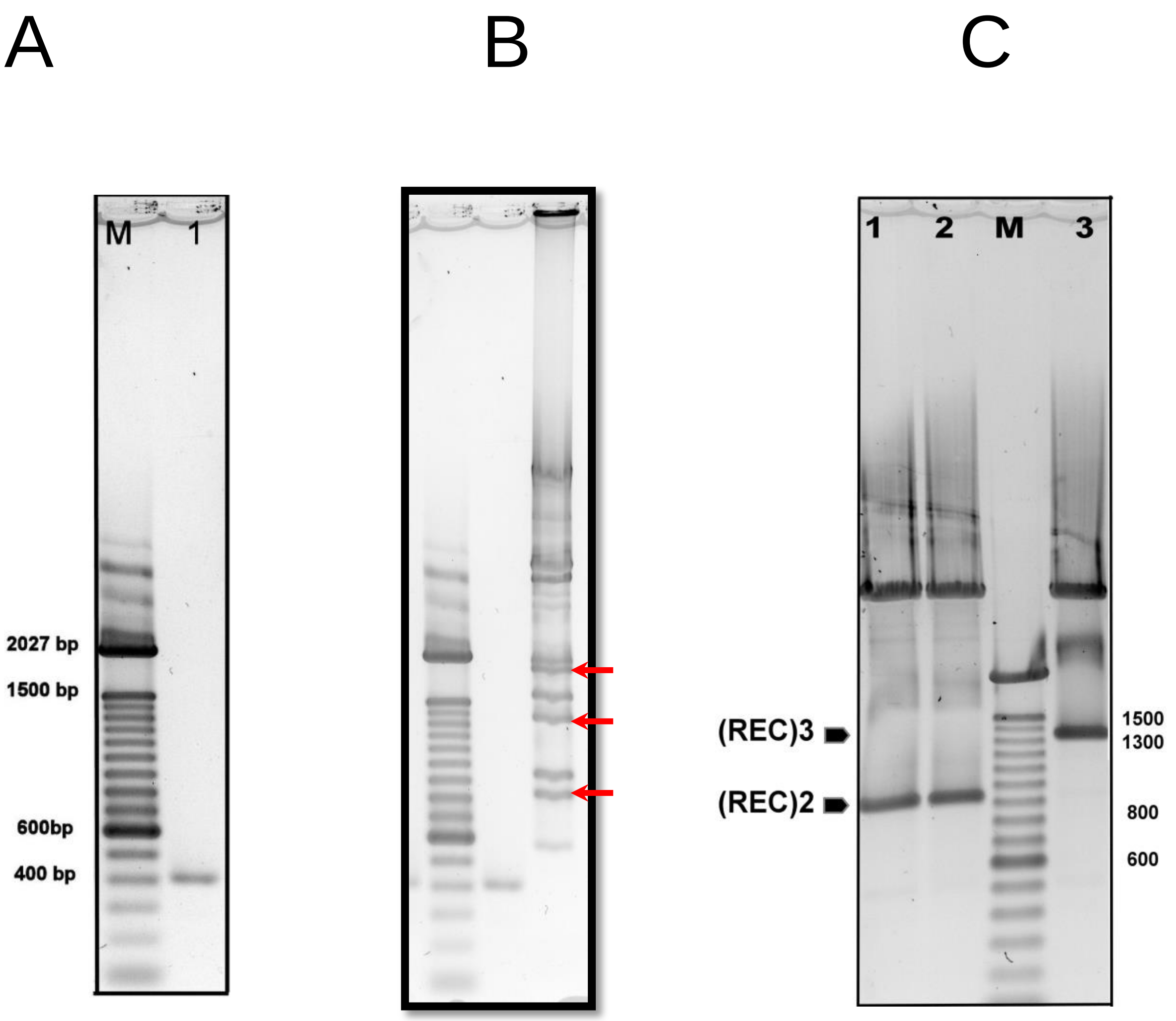

Figure S3: 1% agarose gels electrophoresis of: monomer gene (M-REC) PCR mutagenesis (A), M-REC concatamerization (B) diagnostic digestion of randomly selected colonies plasmids (C). Panel A, Lane1: PCR amplification product using *Sap*IREC for and *Ear*IREC rev primers. The band has the expected molecular weight of the mutation product, which is the restriction sites of endonucleases flanking the M-REC. Type IIS restriction enzymes *Ear*I and *Sap*I allowed seamless and unidirectional cloning avoiding extraneous amino acid residues within the sequence of the recombinant polymer Panel B, Lane 1: *Ear*I digested monomer gene. Lane 2: Concatamerization products. The red arrows mark dimeric (n=2), trimeric (n=3) and tetrameric (n=4) M-REC concatenamers. Panel C , Lanes 1-2-3 show the *Eco*RI restriction analysis of pDrive vector containing multimeric REC genes. *Eco*RI's digestion releases the cloned fragments plus 100 bp of plasmid sequence (3750 bp). The result shows fragments whose size is compatible with dimer (lanes 1-2) and trimer clones (lane 3) respectively. The position of the bands corresponding to the genes of interest are highlighted with arrow. Lane M: DNA molecular weight marker, the molecular weight of some bands is indicated.

pET 10 6K

```
                                                                M  G  K  K  K  P  V
1      CCCCTCTAGAAATAATTTTGTTTAACTTTAAGAAGGAGATATACATATGGGTAAAAAAAAGCCGGTAGGAAGAGCTGGTC   80
1      GGGGAGATCTTTATTAAAACAAATTGAAATTCTTCCTCTATATGTATACCCATTTTTTTTCGGCCATCCTTCTCGACCAG   80
           XbaI                                                 NdeI           SapI EarI

                                                                  V  K  K  K STOP
81     TGCTGCTCCTCGCTGCCCAGCCGGCGATGGCCATGGATATCGCTCTTCAGTAAAAAAGAAATGAGAGCTCCGTCGACAAG   160
81     ACGACGAGGAGCGACGGGTCGGCCGCTACCGGTACCTATAGCGAGAAGTCATTTTTTCTTTACTCTCGAGGCAGCTGTTC   160
                                                        SapI EarI

161    CTTGCGGCCGCACTCGAGATCAAACGGGCTAGCCAGCCAGAACTCGCCCCGGAAGACCCCGAGGATGTCGAGCACCACCA   240
161    GAACGCCGGCGTGAGCTCTAGTTTGCCCGATCGGTCGGTCTTGAGCGGGGCCTTCTGGGGCTCCTACAGCTCGTGGTGGT   240
                   XhoI
```

pET 14 8H

```
         N  P  L  E  Y  F  V  Y  F  R  R  R  Y  T  M  G  S  S  H  H  H  H  H  H  H  H
1      GAATCCTCTAGAATATTTTGTTTACTTTAGAAGGAGATATACCATGGGCAGCAGCCATCATCATCATCATCACCACCACG   80
1      CTTAGGAGATCTTATAAAACAAATGAAATCTTCCTCTATATGGTACCCGTCGTCGGTAGTAGTAGTAGTAGTGGTGGTGC   80

       G  L  V  P  R  G  S  H  M  L  E  D  P  A  A  N  K  A  R  K  E  A  E  L  A  A  A
81     GCCTGGTGCCGCGCGGCAGCCATATGCTCGAGGATCCGGCTGCTAACAAAGCCCGAAAGGAAGCTGAGTTGGCTGCTGCC   160
81     CGGACCACGGCGCGCCGTCGGTATACGAGCTCCTAGGCCGACGATTGTTTCGGGCTTTCCTTCGACTCAACCGACGACGG   160
                         NdeI   XhoI  BamHI
```

Figure S4. Nucleotide and amino acid sequences of the codifying region of the vectors pET10 6K (above) and pET 14 H8 (below). The modified regions of the original vectors are highlighted in green while the position of the restriction sites of the endonucleases used in the subcloning is indicated in bold.

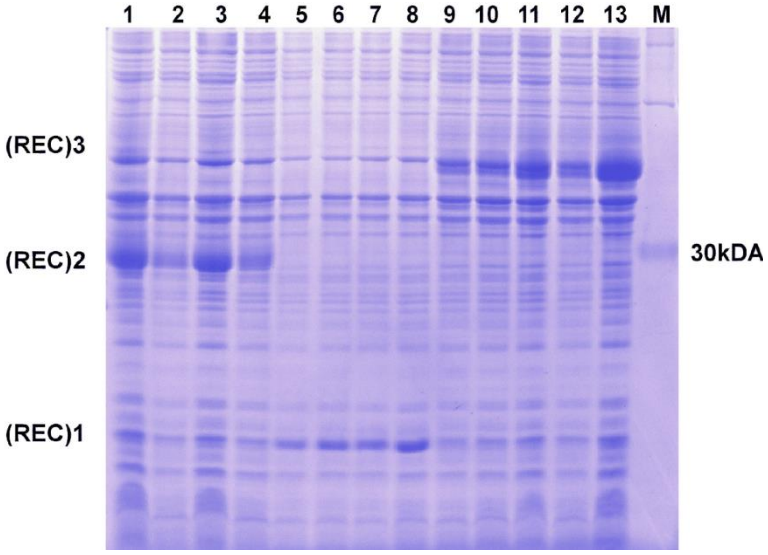

Figure S5. 15% SDS-PAGE Comassie-blue stained of total protein fractions of *E. coli* BL21(DE3) strain colonies after overnight induction in modified TB medium. 13 transformed colonies were randomly selected and screened: lanes 1 to 4 -(REC)2 theoretical MW 23926.4 Da; lane 5 to 8 (REC)1 theoretical MW 12549.1 Da; lanes 9 to 13 (REC)3 theoretical MW 35303.8 Da. Lane M: protein molecular weight marker, whose 30kDa band is indicated.

MGSSHHHHHHHHGLVPRGSHMGKKKPV**(**SDTYGAPGGGNGGRPSDTYGAPGGGNGGRPS

DTYGAPGGGNGGRPSDTYGAPGGGNGGRPGLGGVGLGGVGLGGVGKLGGVGLGGVGLG

GVGLGGVGPKGPGGPGGPGGPGGPGGPKGPAGPAGPAGPAGPAGPKGV**)$_3$** KKKV

Figure S6: Amino acid sequences of (REC)$_3$ polypeptide (one letter code).

Amino acid analysis

	AA	N measured	N theoretical
D	ASP	12,54	12
E	GLU		
N	ASN	12,54	12
S	SER	16,46	15
Q	GLN		
H	HIS	11,84	9
G	GLY	188,12	196
T	THR	12,31	12
R	ARG	14,42	13
A	ALA	25,16	27
Y	TYR	12,54	12
C	CYS		
V	VAL	23,51	27
M	MET	2,27	2
W	TRP		
F	PHE		
I	ILE		
L	LEU	20,46	22
K	LYS	17,33	18
P	PRO	66,62	65
		436,1	442

Figure S7. Amino acid analysis of the $(REC)_3$ polypeptide. Differences among experimental and theorical amino acid number and composition values were assigned to the experimental error associated with the technique. The accuracy of about 5% is dependent on the amino acidic residue.

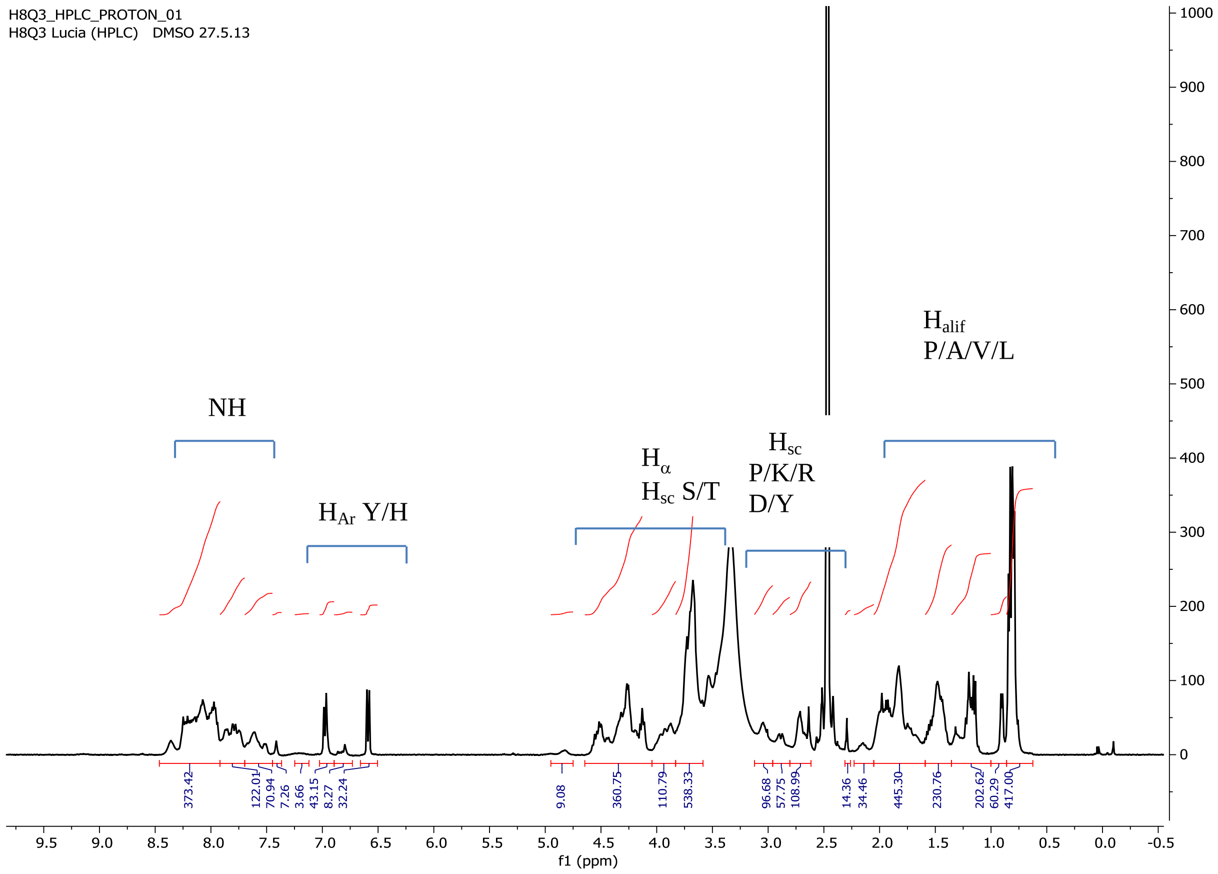

Figure S8. ^{1}H-NMR spectrum of $(REC)_3$ polypeptide recorded in DMSO-d_6

The ^{1}H-NMR spectrum of $(REC)_3$ polypeptide shows signals at different chemical shifts corresponding to different protons belonging to $(REC)_3$ polypeptide. In fact, the chemical shifts of the amide protons are in the range 8.5-7.2 ppm, whereas in the aromatic range (7.1-6.4 ppm), we can recognize the chemical shifts arising from histidine and tyrosine. In the range of 3.5-0.6 ppm we observe signals, assigned to aliphatic side chains of amino acids such as valine, proline, leucine and alanine residues (2.4-0.6 ppm) as well as other amino acids such as lysine, serine, asparagine, arginine and aspartate residues (3.5-2.5 ppm). The signals in the range between 3.5 and 4.6 ppm belong to H_α protons of the amino acids as well as to H_β protons of serine and threonine residues.

MALDI-TOF analysis

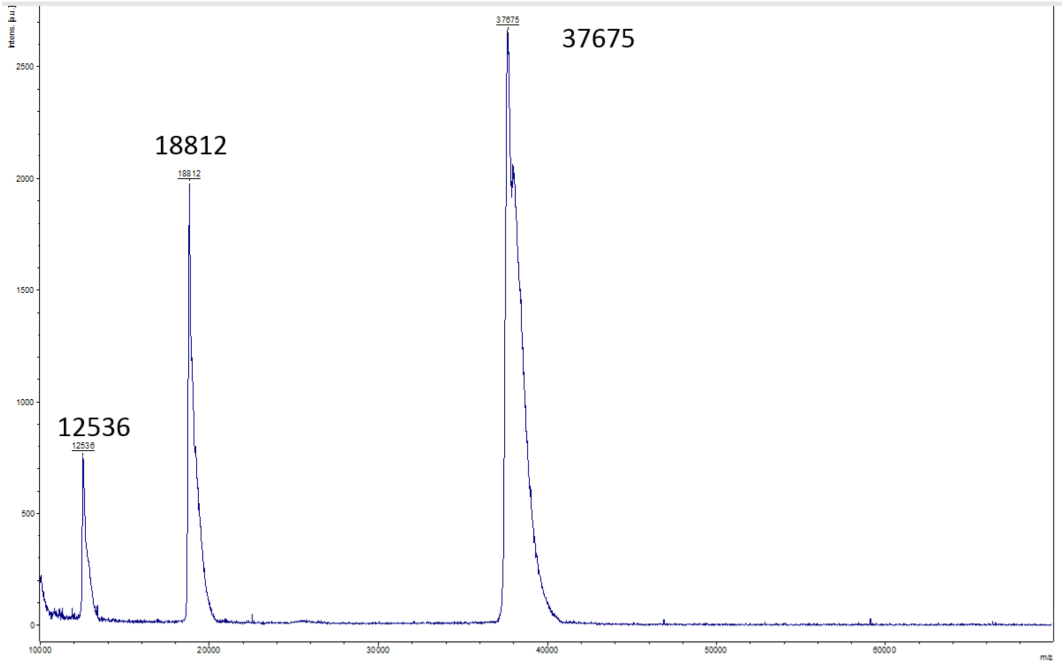

Figure S9. MALDI-TOF of $(REC)_3$ polypeptide. MS spectrum shows the first three ionization states of the molecule.

Table S1. MALDI-TOF spectra analysis of the $(REC)_3$ polypeptide.

**$(REC)_3$**	**Ionization State**	**Theoretic Mass (Da)**	**Experimental Mass (Da)**
	**M^{1+}**	**37660**	**37675**
	**M^{2+}**	**18830**	**18812**
	**M^{3+}**	**12553**	**12536**

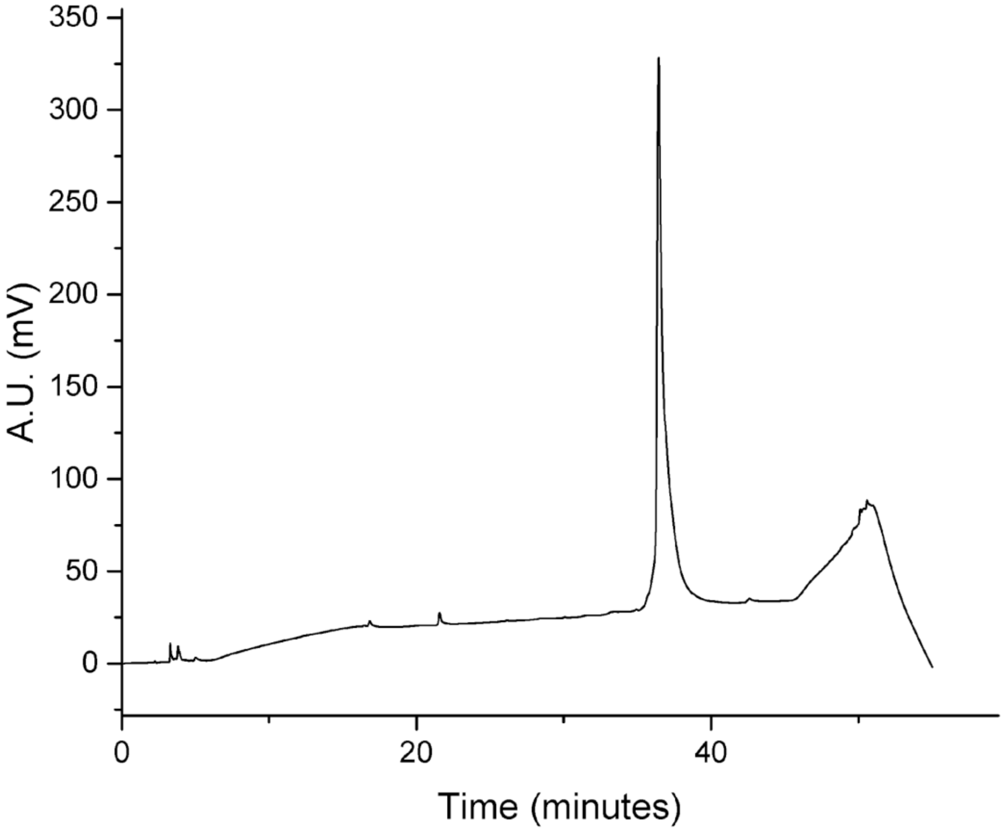

Figure S10: Chromatogram of $(REC)_3$ polypeptide after RP-HPLC purification.

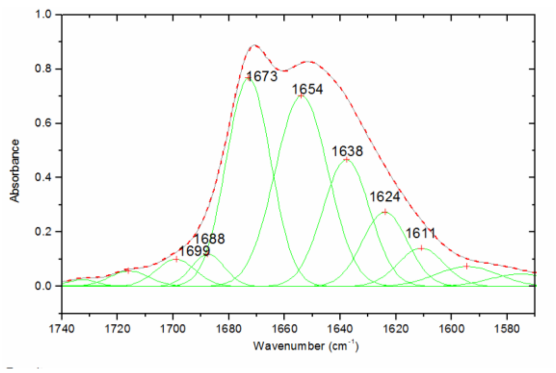

Figure S11. Decomposed FT-IR spectrum of $(REC)_3$ polypeptide amide I region in D_2O solution. The band fitting results of amide I and II regions are shown. Dashed line: experimental spectrum. Solid line: calculated spectrum.

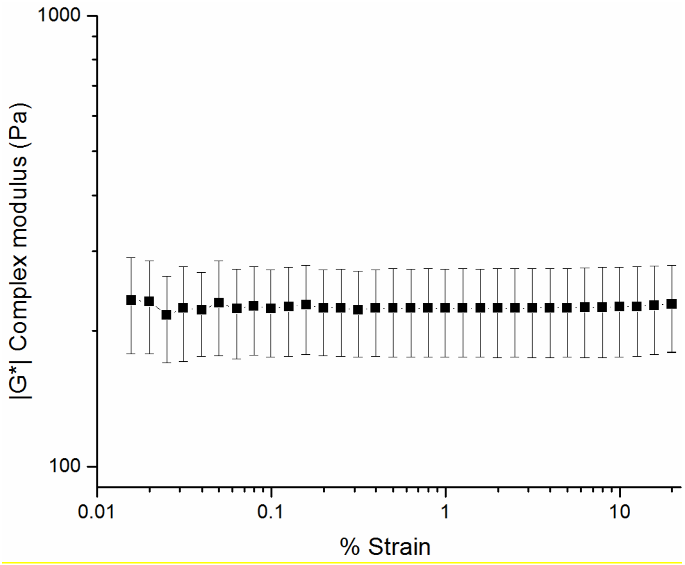

Figure S12: Strain dependence of the complex modulus magnitude, |G*|, at 37°C for $(REC)_3$ hydrogel at a concentration of 50 mg/mL.

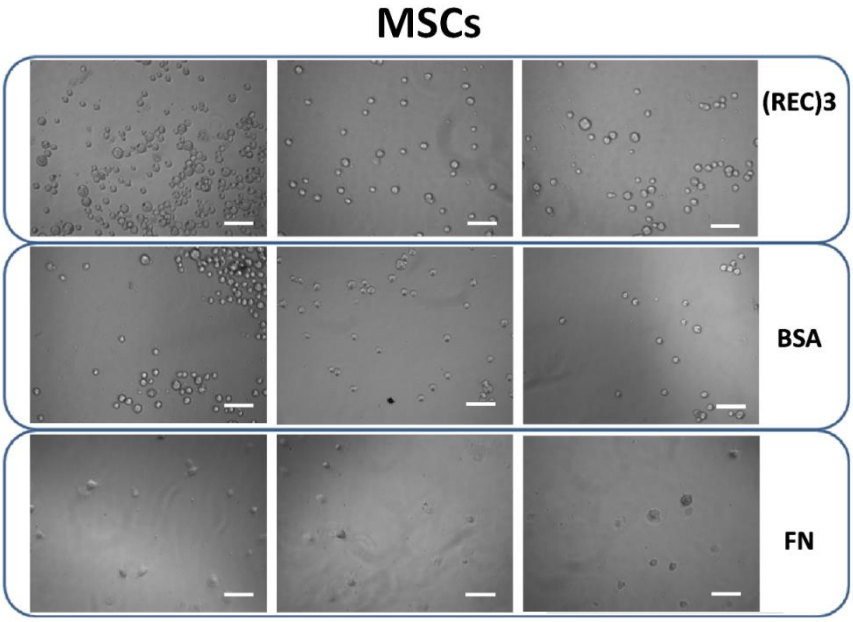

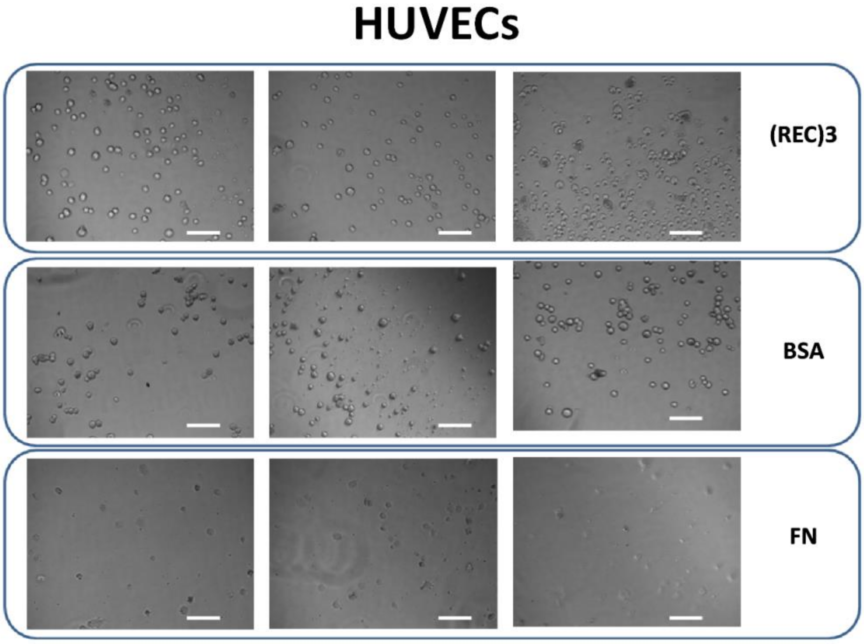

Figure S13. Representative phase-contrast images of mesenchymal (MSCs) and endothelial (HUVECs) cells seeded on protein-coated surfaces. Early adhesion of MSCs and HUVEC after 1 hour of incubation. From top to bottom: MSCs incubated on $(REC)_3$; BSA (negative control); fibronectin (FN) (positive control); HUVECs incubated on: $(REC)_3$; BSA (negative control); fibronectin (FN) (positive control). Pictures were taken at $10\times$ objective magnification and the bars correspond to 100 μm. Both cell types acquire a spread morphology with increased cell areas when incubated on positive control surfaces. In contrast, cells incubated on negative control and $(REC)_3$ coated surfaces show a higher intensity of brightness (parameter dependent on cell-surface distance) and globular shape typical of non-adherent cells.

(A. Pierres et al. Cell Membrane Alignment along Adhesive Surfaces: Contribution of Active and Passive Cell Processes, Biophysical Journal, 2003 84, 2058–2070)
